# Supplementary figures and images for: Dynamical Patterns of Cattle Trade Movements
Source: PLoS One. 2011 May 18;6(5):e19869. doi: 10.1371/journal.pone.0019869 (PMC3097215; doi:10.1371/journal.pone.0019869)

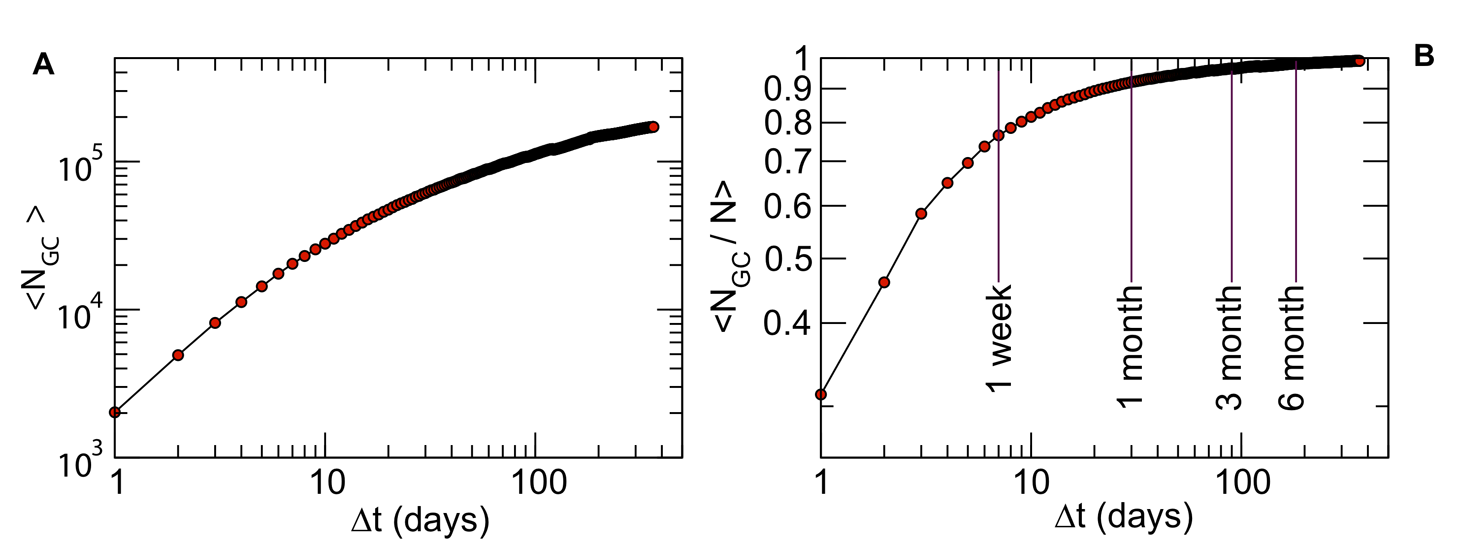

Supplement: Figure S1 — Size of the giant component of the network for increasing aggregation timescale . Average number of nodes (panel A) and relative fraction with respect to the system size (panel B) of the giant component of networks aggregated on time windows of length . As increases, the networks become more globally connected. Sizes are averaged over all snapshots obtained with a given value of . (TIFF) [file pone.0019869.s001.tiff]

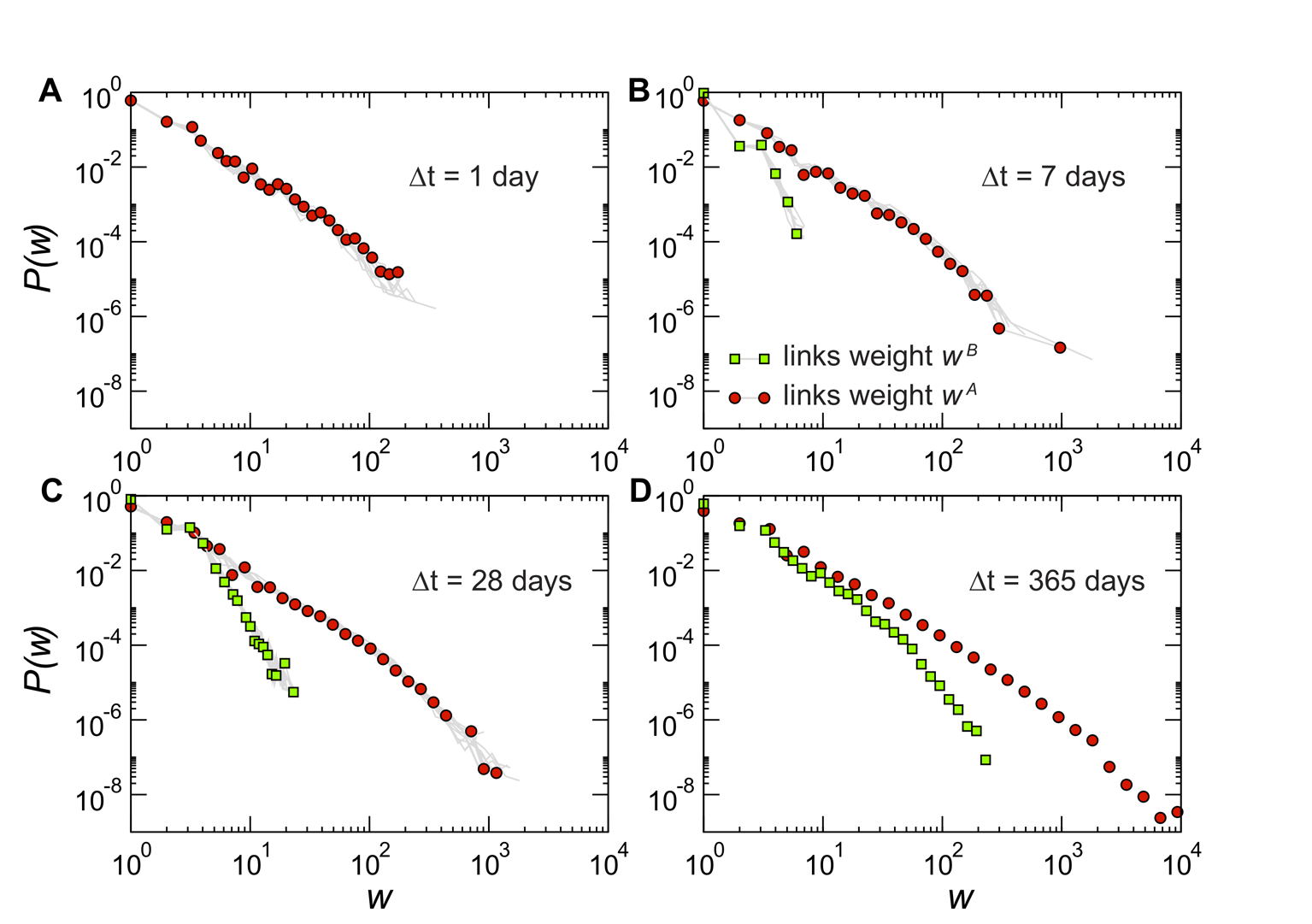

Supplement: Figure S2 — Weight distributions for networks aggregated on different timescales . Red circles refer to the binned distributions of the weight , measuring the number of animals moved along the link ij, whereas green squares refer to the binned distributions of the weight that counts the number of batches displaced along the link. The same representation of Figure 1 of the main text is adopted, with symbols representing the result of a particular snapshot, and grey lines the results obtained for a subset of the other snapshots. The cut-off of the distributions is naturally fixed by the choice of the aggregating period . The distribution of for the daily networks has been omitted, since it is equal to 1 for and 0 elsewhere. (TIFF) [file pone.0019869.s002.tiff]

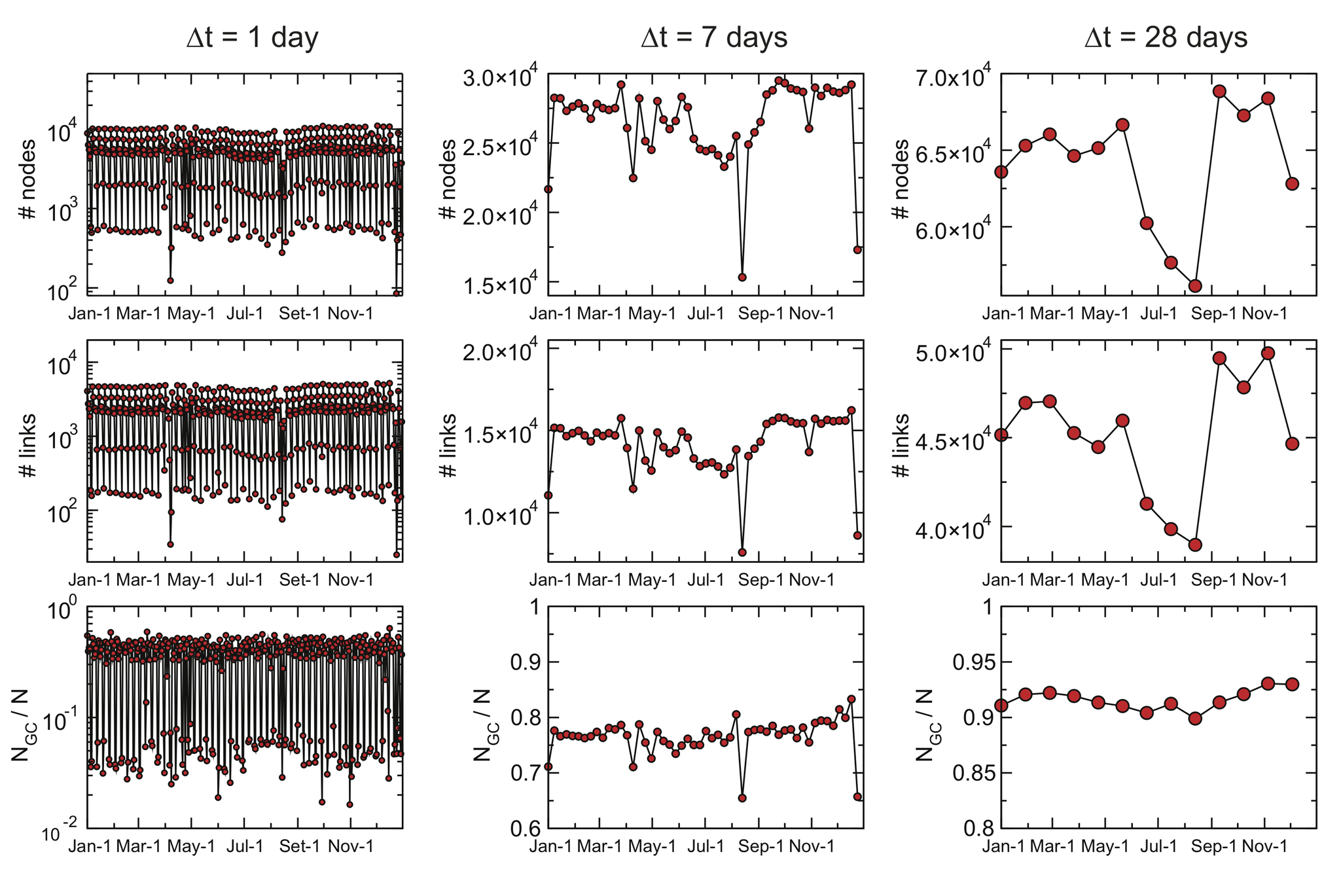

Supplement: Figure S3 — Time evolution of the global static features of networks on different timescales. The timeline of the number of nodes (top), the number of links (center), and the fraction of nodes in the giant component (bottom) are shown for daily, weekly, and monthly networks. Clear weekly and seasonal patterns are detected. (TIFF) [file pone.0019869.s003.tiff]

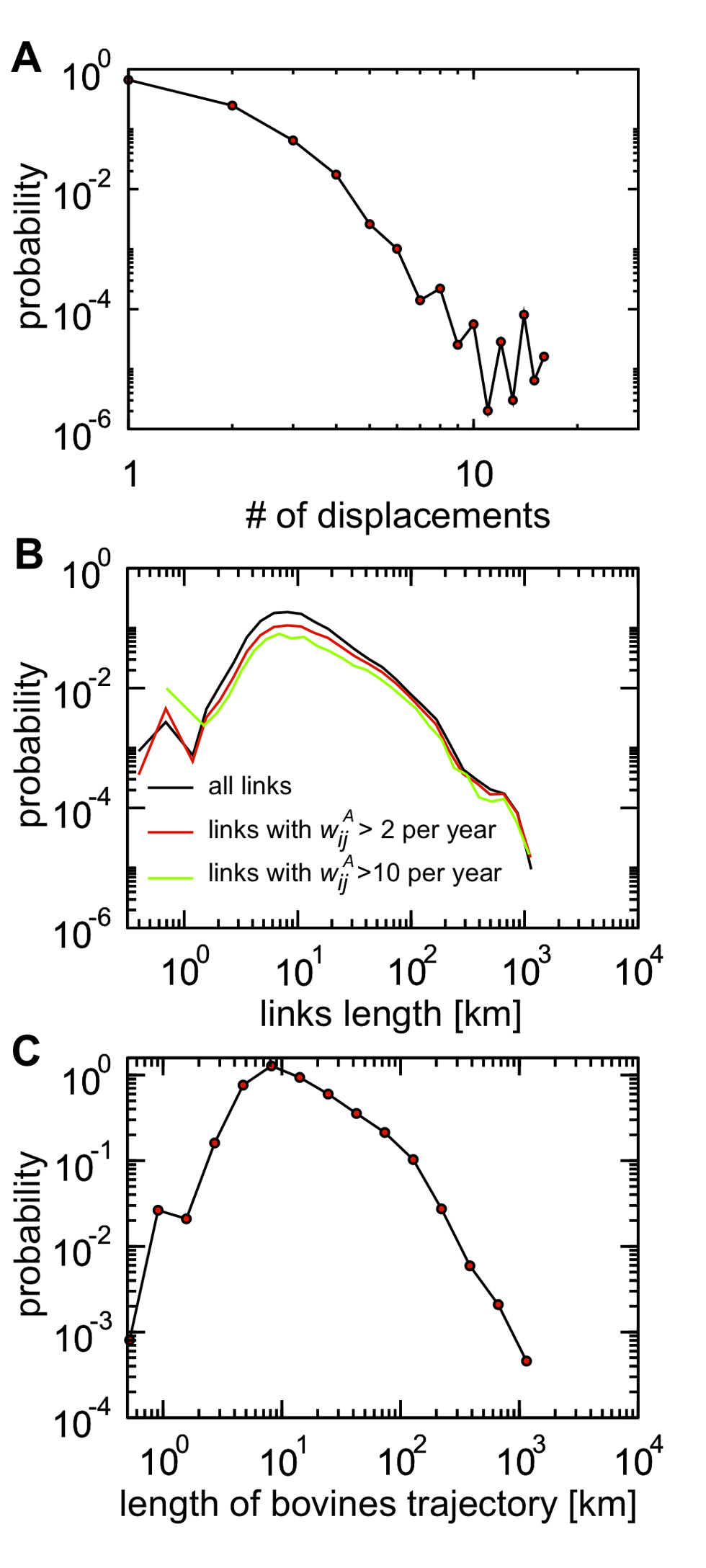

Supplement: Figure S4 — Bovine activity. Panel A shows the probability distribution of the number of displacements that a bovine experiences during one year. Panel B displays the probability distributions of the distances covered during a single displacement. Since many links correspond to the displacement of very few animals, the same distribution is shown with different thresholds, i.e. considering only links with at least 2 or 10 bovines displaced during the year under study. This corresponds to keeping respectively 42% and 13% of the original links. Panel C shows the probability distribution of the distances covered by a single animal during its trajectory in one year. (TIFF) [file pone.0019869.s004.tiff]

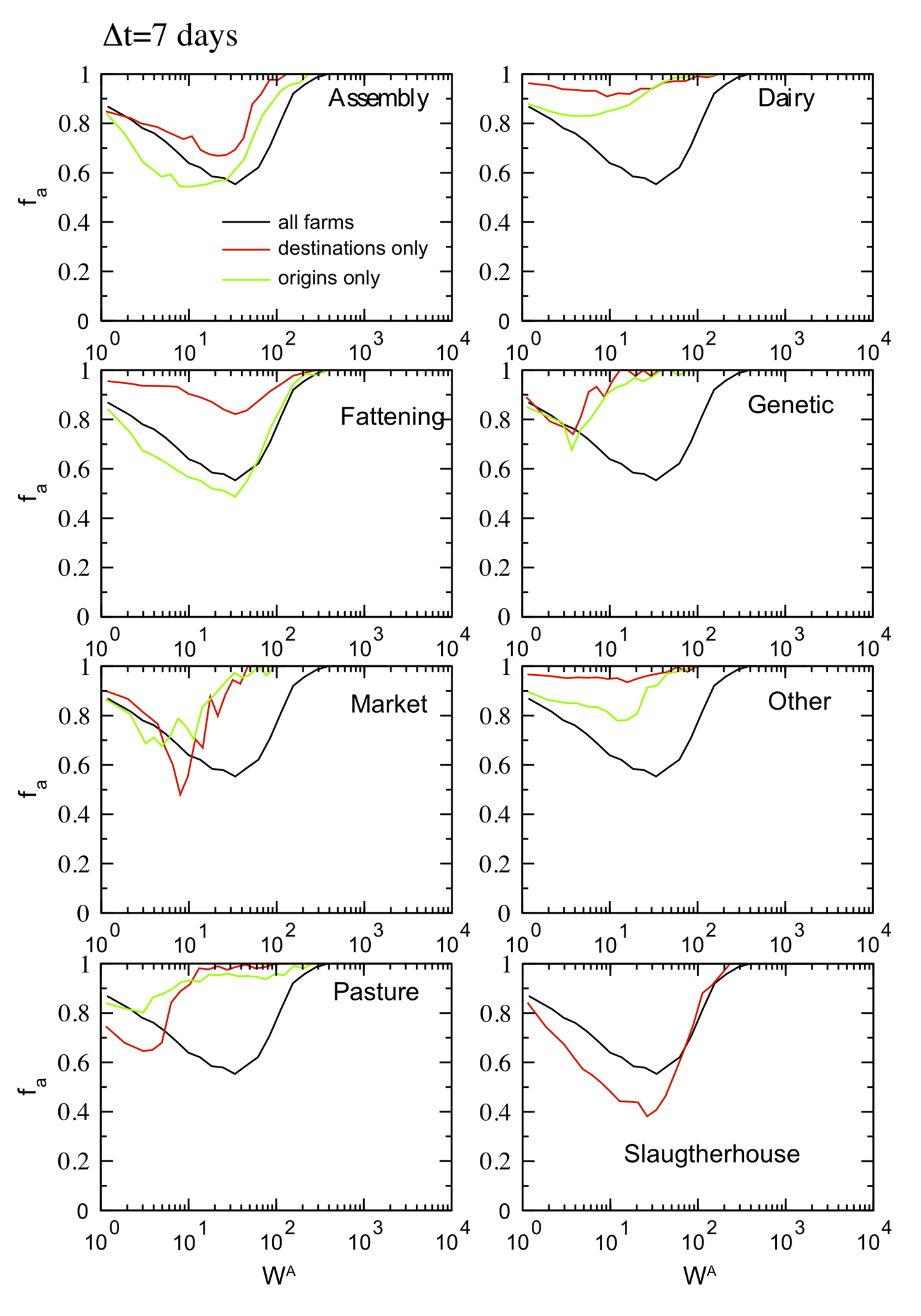

Supplement: Figure S5 — Appearance of nodes by premises type. The fraction of appearing links as a function of the weight associated to the link. The black curve refers to the total fraction of appearing links, the red curves are obtained considering only the links pointing to a given premises type, the green curves are obtained using only the links originating at a given premises type. The links’ behavior depends on the nature of the displacement, i.e. the premises type of origin/destination. Consistent results are obtained for different timescales . The fraction of disappearing links (not shown) displays an almost identical behavior (similarly to the results presented in Figure 7 of the main text). (TIFF) [file pone.0019869.s005.tiff]
